# Supplementary figures and images for: Using a topic model to map and analyze a large curriculum
Source: PLoS One. 2023 Apr 20;18(4):e0284513. doi: 10.1371/journal.pone.0284513 (PMC10118121; doi:10.1371/journal.pone.0284513)

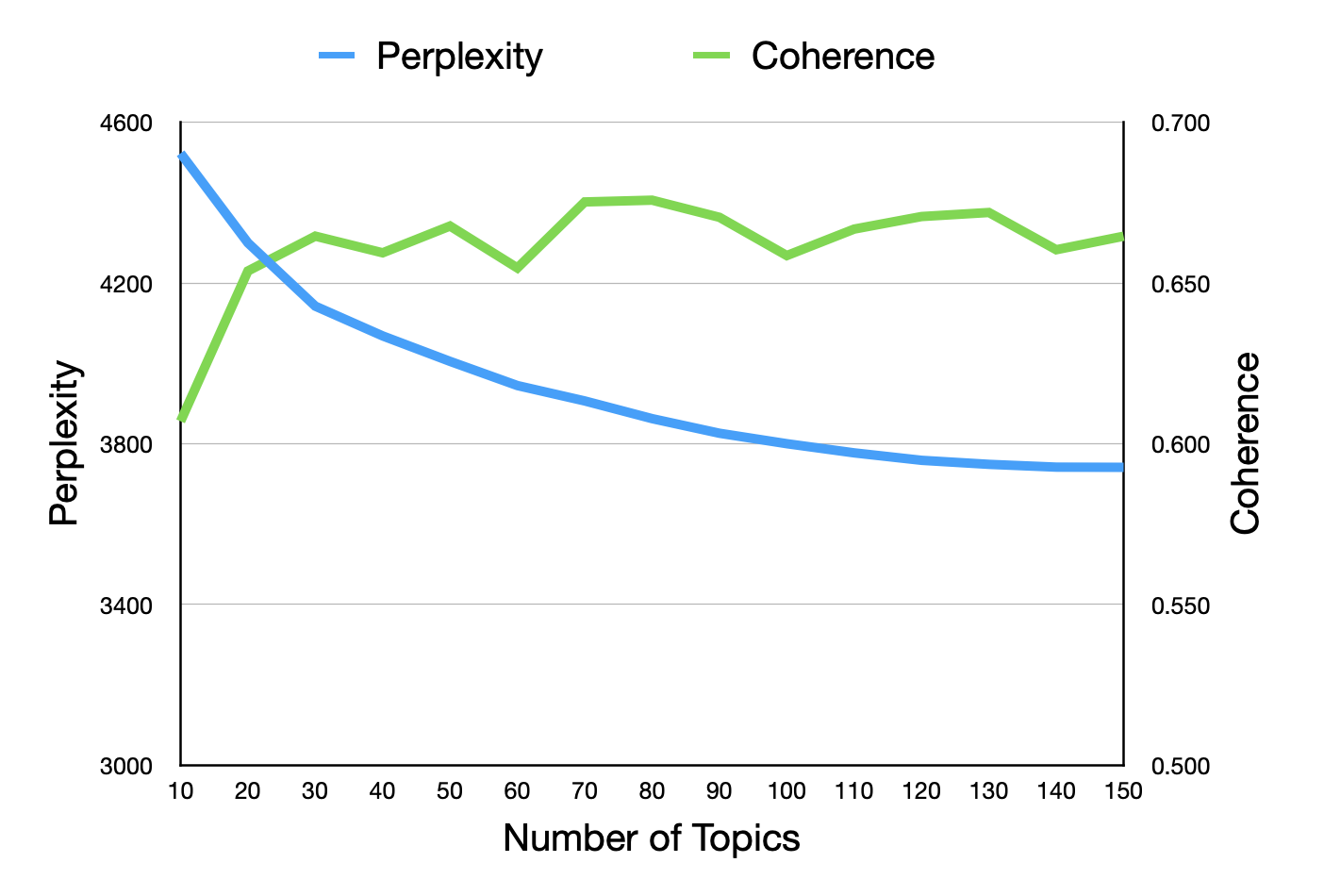

Supplement: S1 Fig — LDA Mallet was used to generate topic models for texts from the class of 2024. Models were generated for topic numbers from 10 to 150 at increments of 10. The quality of the model at each topic number was evaluated by topic coherence and perplexity on a held-out set of texts. Models with higher topic coherence scores have been found to generate topics that make more sense to human reviewers. Models with lower perplexity scores more accurately predict words in an unseen set of texts. (TIF) [file pone.0284513.s001.tif]

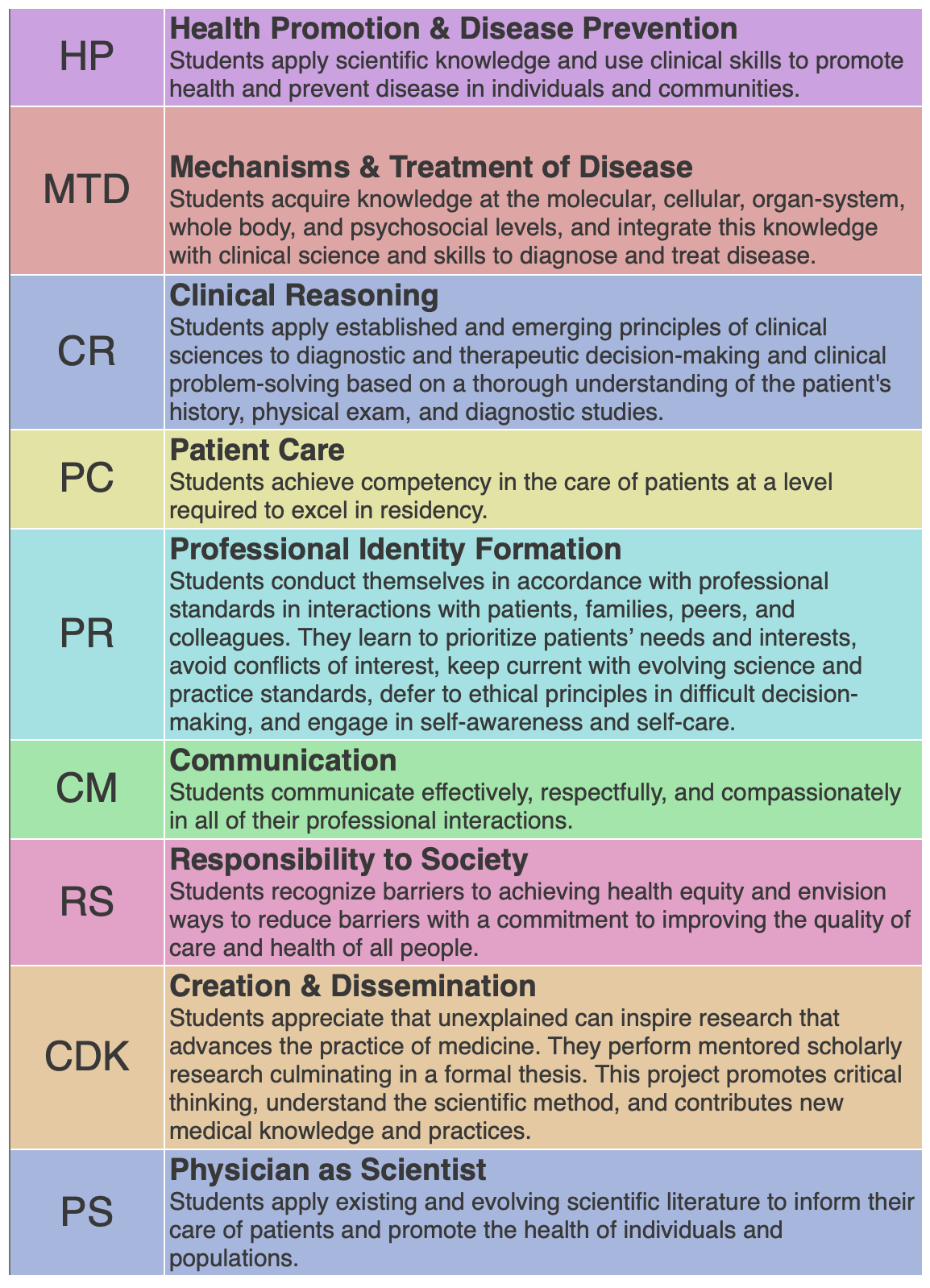

Supplement: S2 Fig — A list of nine competencies that students are expected to meet before graduating. A major goal is to map content in the curriculum to these competencies. (TIF) [file pone.0284513.s002.tif]

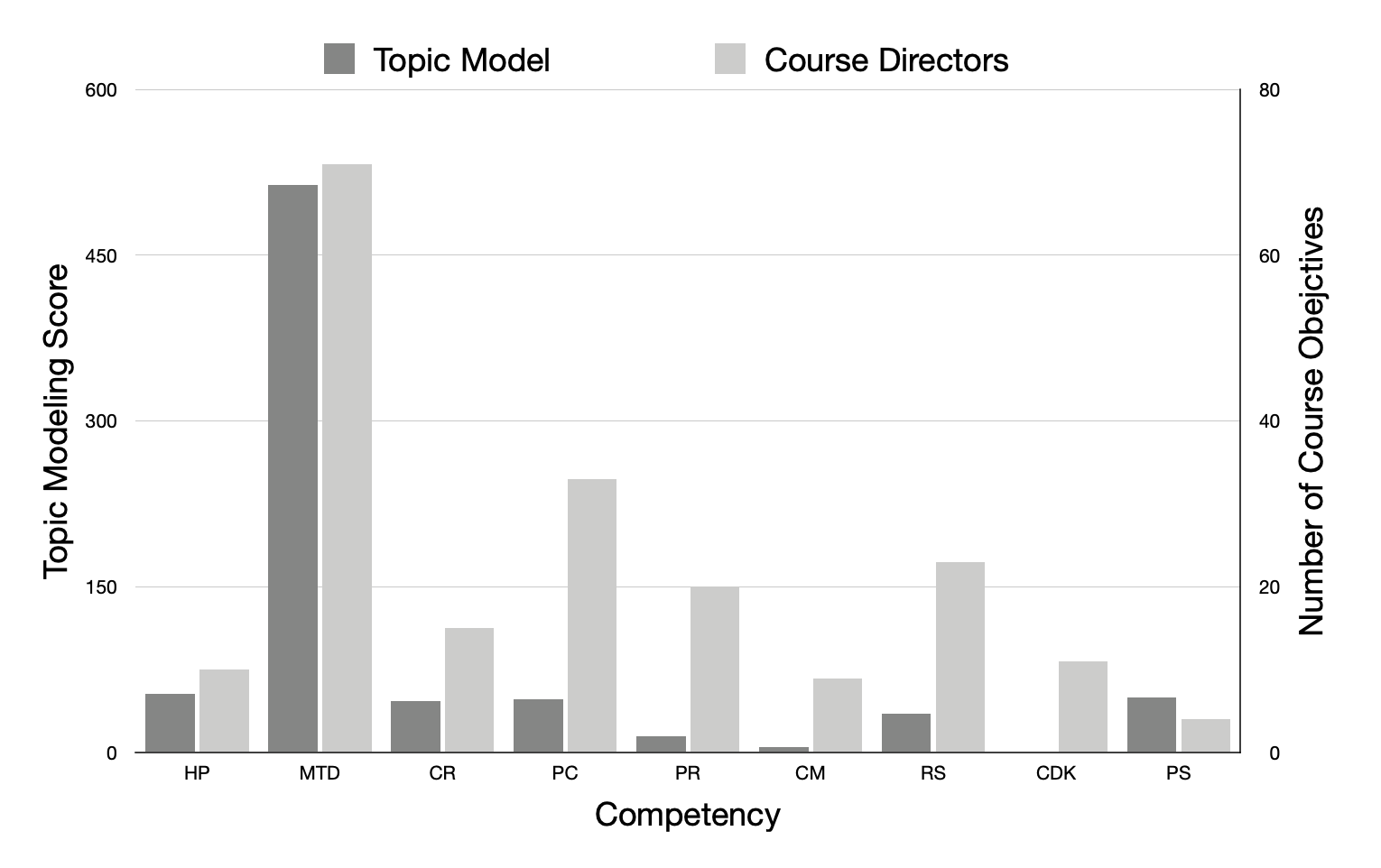

Supplement: S3 Fig — The mapping of content to competency is the same as in Fig 2. Superimposed is the mapping of course objectives to competencies performed by the course directors. Each course director mapped the objectives for their course to the competencies. The results show the mapping combined from all the courses in the pre-clerkship curriculum. (TIF) [file pone.0284513.s003.tif]
