# Supplementary material for: Using a topic model to map and analyze a large curriculum
Source: PLoS One. 2023 Apr 20;18(4):e0284513. doi: 10.1371/journal.pone.0284513 (PMC10118121; doi:10.1371/journal.pone.0284513)
Supplement: Attachment — Submitted filename: Response to Referees.docx [file pone.0284513.s004.docx]

I thank the reviewers for their insightful comments and suggestions. I have submitted a revised manuscript that addresses their concerns. Below I detail the changes in the manuscript that were made in response to each of their comments.

Response to Reviewer Comments

Reviewer #1: Dear Authors,

Q1. This type of work is very difficult for me to assess as I believe that there is a great deal of value in it, but it does not argue for a specific claim other than that it is possible to apply a certain method to a data set. With respect to this claim you have done an excellent job. The LDA method and subsequent analyses have been applied and explained in depth. However, the validity of the measures (by which I mean, do they mean what you say they mean) has not been investigated. The metrics you report cannot be compared to metrics from anywhere else, for example another corpus of texts. You have not user-tested the metrics you developed so cannot characterize whether they would be useful in course review or construction, or whether there are any unforeseen negative consequences for their use. This does not mean that your research is not valuable, it is a necessary step towards developing new metrics. The question is whether it is publishable on its own, or whether the publication also needs some attempt to validate the approach. I don't believe there is consensus on this, and I have heard very strong opinions on both sides. Unfortunately I don't really know what the answer is though but you should acknowledge the issue and how it could be approached. Network metrics have long been suggested as tools to investigate curricula (see anything by Shane Dawson or Caroline Haythornthwaite) but they never see widespread adoption.

**Response:** I recognize the referee’s concern about validating the model. As an initial step toward validating the model, I compared the mapping of content to competency generated by the topic model to mapping of course objectives to competencies done by course directors. This is not a direct comparison because course objectives could encompass different amounts of content, but the results show similar distributions of mapping to competencies between the topic model and what was produced by course directors. The results are shown in Supplemental Figure 3 and described in the Results section. The Conclusion also describes the need to further validate the model.

There are also some methodological pieces that could use clarification.
1. The exact way that the topics were mapped to the competencies. Was this a manual process? If so, was it done by a single individual or by several and if by several do you have inter-rater reliability measures?

**Response:** The topics were manually mapped to the competencies by the author. This information was added to the Methods section.

2. The number of standard deviations from the mean used to determine relatedness of the sessions needs some justification. Four seems like a lot to me, but it may not be, you should say why you chose 4.

**Response:** The cutoff score was determined empirically, Network graphs were generated for cutoff scores 2, 3, 4, or 5 standard deviations below the mean. I reviewed about 20% of the connections between sessions for which I have detailed knowledge of the content. From this review, I determined that a cutoff score 4 standard deviations below the mean generated the most accurate network graph. This information has been added to the methods and results sections.

Q3. I am not sure to what format would be appropriate to attach you data. The dashboards you have provides through links are likely insufficient unless I am reading them wrong. To be in compliance with the PLOS Data Policy you would likely need to provide access to a minimally processed corpus.

**Response:** Corpuses for the four curricular years have been made publicly available here:

<https://doi.org/10.7910/DVN/CFH8RO>

Q4. The manuscript will require substantial copy-editing, there are many typos.

**Response:** The revised manuscript has been thoroughly reviewed for typos.

Reviewer #2: It is a well written manuscript with a good attention to details. After reading it with interest, I think this manuscript would need a minor revision to address two points that would improve the robustness of the paper:
1. Please provide cross-validation details for your TMs in the methodology and result/supplementary material. It is crucial that TMs are cross-validated as you used an unsupervised version of TMs.

**Response:** Cross validation was performed for models with topic numbers from 10 to 150 at increments of 10. The results are shown in Supplemental Figure 1.

2. I think a lot of critical inference is being missed without a conclusion section. Please write a conclusion section to present the implication of this study in shaping higher-education policies that can be applied beyond YSM.

**Response:** A Conclusion section has been added to the revised manuscript and the implications of the study on shaping

3. Some context why MTD has such s significantly high competence score (Figure 2) can be helpful in improving the readability to a broader audience. I think the paper would highly benefit from a table that devise all the curriculum jargons with some contextualised examples.

**Response:** Because the pre-clerkship curriculum was analyzed, most of the topics were expected to map to MTD (Mechanism and Treatment of Disease). Most sessions in the pre-clerkship curriculum cover basic or clinical science whose content mostly covers the mechanisms and treatments of disease. This explanation was added to the results sections. I have tried to identify curriculum jargon in the text and more clearly define the jargon in the text, but if there are terms that still seem unclear, I can define those, too. Supplemental Figure 2 lists all the competencies with a brief description of each**.**
